# The Philosophy of Dermatopathology

**DOI:** 10.3390/diagnostics12123091

**Published:** 2022-12-08

**Authors:** Yasuhiro Sakai

**Affiliations:** Department of Joint Research Laboratory of Clinical Medicine, Fujita Health University School of Medicine, Aichi 470-1192, Japan; ya-sakai@fujita-hu.ac.jp; Tel.: +81-562-93-9934

Diagnostic pathology involves studying sample cells and tissues obtained from the specific lesions of interest. It is designed not only to observe changes occurring in actual cells and tissues using morphologic, immunologic, microbiologic, and molecular biologic techniques but also to explain the reasons underlying these changes and ultimately to confirm the diagnosis.

Dermatopathology is one of the most sophisticated areas of diagnostic pathology; we can easily observe superficial skin lesions using only our eyes without invasive techniques such as an endoscopic or operational approach. For example, more than one hundred kinds of “dermatitis” are now being subclassified and studied because dermatologists have been making detailed gross observations of cardinal inflammatory signs, including heat, pain, redness, and swelling, for two thousand years—ever since Aulus Cornelius Celsus first provided descriptions. Henry Seguin Jackson originally coined the term *dermato-pathologia* in 1792, and since then, these visual signs have been compared to microscopic findings.

Thereafter, pathologists such as Rudolph Ludwig Karl Virchow, one of the greatest pathologists in history, paid very little attention to dermatopathology. While dermatopathology was originally developed based on dermatologists’ significant efforts, as a result of this lack of attention, even now many general pathologists barely understand the specialty of dermatopathology, such as the many classifications of cutaneous disorders, pathoetiologic wavelength, clinicopathological relationship, and glossaries unique to dermatopathology.

In order to further promote “dermatopathology”, we should bridge the divide between dermatology and pathology using morphologic, immunologic, microbiologic, and molecular techniques, even if only in small steps. For example, keratoacanthoma is one of the most “divided” cutaneous disorders. It is pathologically difficult to distinguish from well-differentiated invasive squamous cell carcinoma; however, it exhibits a distinct clinical behavior and may regress spontaneously. Ogita and Ansai deepen the morphological consideration and focus on the “large pale pink cells”, which are the key criteria for keratoacanthoma [1]. They sharpen the classification of crateriform tumors, including keratoacanthoma, in the view of both dermatologists and pathologists, which may result in changes to the WHO’s criteria. Another difficult example is mycosis fungoides. Mycosis fungoides, particularly in its erythematous phase, is sometimes pathologically indistinguishable from eczematous dermatitis, a benign inflammatory disorder. Miyagaki summarizes the novel diagnostic tools for early mycosis fungoides: novel immunohistochemical markers, such as thymocyte selection-associated high mobility group box factor; cell adhesion molecule 1; the next-generation sequencing of T-cell receptor genes; and microRNA profiles [2].

Recently, dermatology and pathology have been brought together through molecular biology, and dermatopathology has focused on the study of various cutaneous diseases at the molecular level. Dozens and dozens of these are now being well-researched, including melanocytic nevus, malignant melanoma, extramammary Paget disease, atopic dermatitis, psoriasis, epidermolysis bullosa, and lichen sclerosus (et atrophicus). For example, Morikura and Miyata note that mechanical intermittent compression promotes malignant melanoma progression by melanoma cell proliferation and collagen degradation [3]. Acral melanomas in non-sun-exposed skin, such as planta may be associated with mechanical stress. Ohmori et al. show that dermcidin, which is expressed in normal eccrine glands and provides antimicrobial action in sweat, is expressed in Paget cells and is closely associated with a poor prognosis in extramammary Paget diseases [4]. Moniaga et al. review the molecular mechanism of atopic dermatitis. Neuroimmune crosstalk by cytokines associated with type 2 inflammation, such as interleukin (IL)-4, IL-5, IL-13, and IL-31, which stimulates cutaneous sensory neurons and causes itching [5]. Kishimoto et al. note that STAT3 is not closely related to extracutaneous cancers in patients with psoriasis, although STAT3 is activated in psoriatic cutaneous lesions as well as multiple cancerous tissues [6]. Tawfik et al. demonstrate that various single-nucleotide polymorphisms in the PSORS1 locus are significantly associated with psoriasis in an Egyptian cohort [7]. Pânzaru et al. review the clinical and genetic heterogeneity of epidermolysis bullosa and summarize the genotype–phenotype correlation [8]. Oyama and Hasegawa review the dermatophysiology and functional importance of extracellular matrix protein 1 (ECM1) and explain the etiopathological relationship between ECM1 and lichen sclerosus [9].

Dermatopathology is an academic discipline which systematizes human skin diseases by unifying dermatology and pathology, and we should continuously add new and ever-evolving knowledge into the system of dermatopathology. We should also seriously consider reorganizing the system of dermatopathology because molecular biology is so rapidly developing. This Special Issue aims to focus on advances in diagnostic dermatopathology from histopathologic to molecular studies, and we hope that it serves as a trigger to promote the study of dermatopathology.
